# NEURAPRO: a multi-centre RCT of omega-3 polyunsaturated fatty acids versus placebo in young people at ultra-high risk of psychotic disorders—medium-term follow-up and clinical course

**DOI:** 10.1038/s41537-018-0052-x

**Published:** 2018-06-25

**Authors:** B. Nelson, G. P. Amminger, H. P. Yuen, C. Markulev, S. Lavoie, M. R. Schäfer, J. A. Hartmann, N. Mossaheb, M. Schlögelhofer, S. Smesny, I. B. Hickie, G. Berger, E. Y. H. Chen, L. de Haan, D. H. Nieman, M. Nordentoft, A. Riecher-Rössler, S. Verma, A. Thompson, A. R. Yung, P. D. McGorry

**Affiliations:** 1 Orygen, the National Centre of Excellence in Youth Mental Health, Melbourne, Australia; 2 Centre for Youth Mental Health, University of Melbourne, Melbourne, Australia; 3 Department of Psychiatry, Medical University of Vienna, Vienna, Austria; 4 Department of Psychiatry and Psychotherapy, Clinical Division of Social Psychiatry, Medical University of Vienna, Vienna, Austria; 5 University Hospital Jena, Jena, Germany; 6 Brain and Mind Research Institute, University of Sydney, Sydney, Australia; 7 Child and Adolescent Psychiatric Service of the Canton of Zurich, Zurich, Switzerland; 8 Department of Psychiatry, University of Hong Kong, Pokfulam, Hong Kong; 9 Academic Medical Center, Amsterdam, The Netherlands; 10 Psychiatric Centre Bispebjerg, Copenhagen, Denmark; 11 Psychiatric University Clinics Basel, Basel, Switzerland; 12 Institute of Mental Health, Singapore, Singapore; 13 Division of Mental Health and Wellbeing, Warwick Medical School, University of Warwick, Coventry, UK; 14 North Warwickshire Early Intervention in Psychosis Service, Coventry and Warwickshire NHS Partnership Trust, Coventry, UK; 15 Division of Psychology and Mental Health, University of Manchester, Manchester, UK; 16 Greater Manchester Mental Health NHS Foundation Trust, Manchester, UK

## Abstract

This study reports a medium-term follow-up of a randomised, double-blind, placebo-controlled trial of omega-3 polyunsaturated fatty acids (PUFA) in ultra-high risk for psychosis (UHR) patients. Primary outcomes of interest were transition to psychosis and symptomatic and functional outcome. A secondary aim was to investigate clinical predictors of medium-term outcome. Three hundred four UHR participants were recruited across 10 specialised early psychosis services in Australia, Asia, and Europe. The intervention consisted of 1.4 g/daily of omega-3 PUFA or placebo, plus up to 20 sessions of cognitive-behavioural case management (CBCM), over the 6-month study period, with participants receiving further CBCM sessions on basis of need between months 6–12. Mean time to follow-up was 3.4 (median = 3.3; SD = 0.9) years. There was a modest increase in transitions between 12-month and medium-term follow-up (11–13%) and substantial improvement in symptoms and functioning between baseline and follow-up, with no differences between the treatment groups. Most improvement had been achieved by end of the intervention. 55% of the sample received mental health treatment between end of intervention and follow-up. Omega-3 PUFA did not provide additional benefits to good quality psychosocial intervention over the medium term. Although most improvement had been achieved by end of intervention the substantial rates of post-intervention mental health service use indicate longer-term clinical need in UHR patients. The post-intervention phase treatment or the longer-term effect of CBCM, or a combination of the two, may have contributed to maintaining the gains achieved during the intervention phase and prevented significant deterioration after this time.

## Omega-3: No benefit to those at risk of psychosis

Omega-3 fatty acids, when given alongside traditional therapies, provide no extra benefits to patients at high risk of developing psychosis. In attempts to validate a previous clinical trial wherein omega-3 reduced the risk of ultra-high-risk patients transitioning to psychosis, Patrick McGorry, of the National Centre of Excellence in Youth Mental Health, Australia, and his team looked to replicate the findings in a larger cohort. Despite improvements in symptoms seen in a medium-term follow-up of 304 patients after ~3.4 years, no extra benefit or reduced transitioning was seen in those additionally receiving omega-3 fatty acids. The bulk of treatment benefit was seen between 0–12 months, but the data indicate a clear need for ongoing patient care. This study also revealed psychopathology score level, anxiety, and societal functioning to be strong indicators of psychosis transition risk.

## Introduction

The first episode of psychosis is typically preceded by a period of initially subtle and relatively non-specific symptoms followed by a prodromal period characterised by sub-threshold positive symptoms and functional difficulties.^1^ We introduced the “ultra-high risk” (UHR) criteria more than two decades ago in order to prospectively identify young people at incipient risk of progression to full-threshold psychosis.^2,3^ A considerable body of research (>1500 studies) has validated the UHR criteria, examined predictors and mechanisms of onset of psychotic disorder within this clinical population, and enabled trials of a range of treatments aimed at improving symptoms and functioning levels, and reducing the risk of progression (transition) to psychotic disorder.^4^

Twelve trials assessing psychosocial and pharmacological interventions, alone or in combination, have been conducted in UHR cohorts. Meta-analyses have shown that these interventions are effective, with an overall risk reduction of transition to psychosis of 54% at 12 months, with a number needed to treat (NNT) of 8.^5–7^ All treatments in these studies appeared to reduce the risk of progression to psychosis, at least during the first 6–12 months. In line with the clinical staging model,^8–12^ during the earliest stage of illness, safer interventions, such as long-chain omega-3 polyunsaturated fatty acids (PUFA) and cognitive behavioural therapy (CBT), are regarded as the preferred option for first-line treatment. CBT has been found to be effective in many, though not all, of the published trials.^13–19^ The most striking result from the trials to date was the finding that omega-3 PUFA were greatly superior to placebo in reducing transition risk, and psychiatric morbidity in general, not only during the treatment period, but also for a prolonged period (median 6.7 years) subsequently.^20,21^ Omega-3 PUFA are safe, provide broad-spectrum health benefits, and represent a simple and relatively inexpensive treatment strategy.

We therefore attempted to replicate this finding from a single site trial in a larger cohort of 304 UHR participants across 10 sites internationally (the “NEURAPRO” study).^22,23^ At the 12-month follow-up, we observed no difference in transition rates between the two groups (*p* = .76), with both groups improving on clinical and functioning measures.^22^ The failure to replicate the original study may have been due the lack of efficacy of omega-3 PUFA in this patient population. However, other possible reasons are that the lower than expected transition rate (~11% across groups) prevented a test of the efficacy of this treatment and that the other treatments received in both groups (cognitive-behavioural case management (CBCM) and antidepressant medication) may have introduced a ceiling effect beyond which omega-3 PUFA, even if effective, could not be shown to confer additional benefit.^22^ CBCM was not provided in the original study and the rate of antidepressant prescription was lower in that study (10% compared to 62% in the current study.) In the current paper, we report a medium-term follow-up of the NEURAPRO study sample in order to determine if there was an increase in the rate of transition to psychotic disorder after cessation of treatment. Clinical and functional outcomes in the two treatment groups, as well as baseline predictors of outcome, were also of interest.

Previous medium-term outcome studies of UHR intervention trials have varied in their findings. The first medium-term follow-up found that the benefits observed at 6 months of combined risperidone and CBT compared to “needs based intervention” did not persist at 3–4-year follow-up.^24^ Morrison and colleagues^14^ reported that cognitive therapy was superior to monitoring over 1 year. However, at 3-year follow-up, cognitive therapy was no longer superior in preventing transition,^15^ although it was found to be associated with a reduced likelihood of being prescribed antipsychotic medication. A study of olanzapine versus placebo showed trend-level benefits for reducing transition rate in favour of olanzapine after 12 months, although the difference did not reach statistical significance.^25^ At 2-year follow-up transition rate did not differ between the two groups.^26^ Bechdolf and colleagues^27^ reported that an integrated psychological intervention was superior to supportive counselling in high-risk patients at 24-month follow-up. Ising and colleagues^18^ reported benefits in favour of CBT compared to treatment as usual at 4-year follow-up, both in terms of transition rate and remission from UHR status. When pooled together, the medium-term outcome studies (24–48 months) have shown benefits in favour of specific interventions compared to comparison conditions, with the transition risk being reduced by 36% and a NNT of 12.^5^

The aim of the current study was to investigate the medium-term outcome (transition rate, symptomatic, and functional outcome) of the NEURAPRO cohort. While the equally positive outcomes of the two treatment groups (low transition rate, symptomatic and functional improvement) achieved at 12-month follow-up may have been due to the CBCM and/or antidepressant medication received in both groups rather than due to the trial intervention (omega-3 PUFA), it was of interest to investigate whether these benefits were maintained over the medium term or whether there was deterioration in clinical outcomes after cessation of treatment. Secondary aims were to investigate baseline clinical predictors of medium-term outcome and the relationship between clinical measures at the end of the intervention period and symptoms/functioning at medium-term follow-up.

## Results

### Study sample—follow-up

**Fig. 1:**
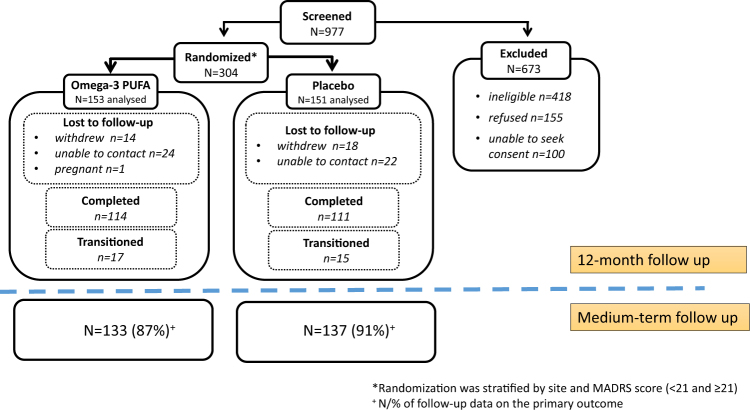
CONSORT diagram of participant distribution

The cohort consisted of 304 participants (153 assigned to omega-3 PUFA treatment, 151 to placebo). Baseline characteristics are presented in McGorry et al.^22^ Medium-term follow-up data were collected for 270 cases (89% of the sample; Fig. 1). eTable 1 presents details of the type of follow-up assessments conducted. Mean time to follow-up was 3.4 years (SD = 0.9 years; median = 3.3 years). The follow-up time range was 1.5–5.7 years (25th percentile = 2.7 years, 75th percentile = 4.0 years). There were no baseline differences on demographic or clinical measures between those who were followed-up vs. those who were lost to follow-up (data available upon request).

One hundred thirty-three participants (55%) had received mental health treatment between end of the study period and medium-term follow-up. These consisted of: general mental health services (*n* = 98, 41%), hospital inpatient admissions (*n* = 26, 11%), UHR services (*n* = 14, 6%), community health services (*n* = 13, 5%), drug and alcohol services (*n* = 4, 2%), forensic services (*n* = 1, 0.4%), and other services (*n* = 42, 18%). Information on pharmaceutical treatment was available for 240 cases (eTable 2).

### Outcomes

#### Primary outcome measure

At the medium-term follow-up, 40 participants (13% of the sample) were known to have transitioned to psychosis. Nineteen of these transitioned cases were from the omega-3 PUFA-treated group (12%) and 21 from the placebo-treated group (14%). Of the 40 transitioned cases, 31 (77.5%) were determined using the CAARMS, 6 (15%) were clinician-determined, and 3 (7.5%) were determined using hospital records. eTable 3 presents the time to transition by treatment group. The maximum time to transition was 4.3 years.

**Fig. 2:**
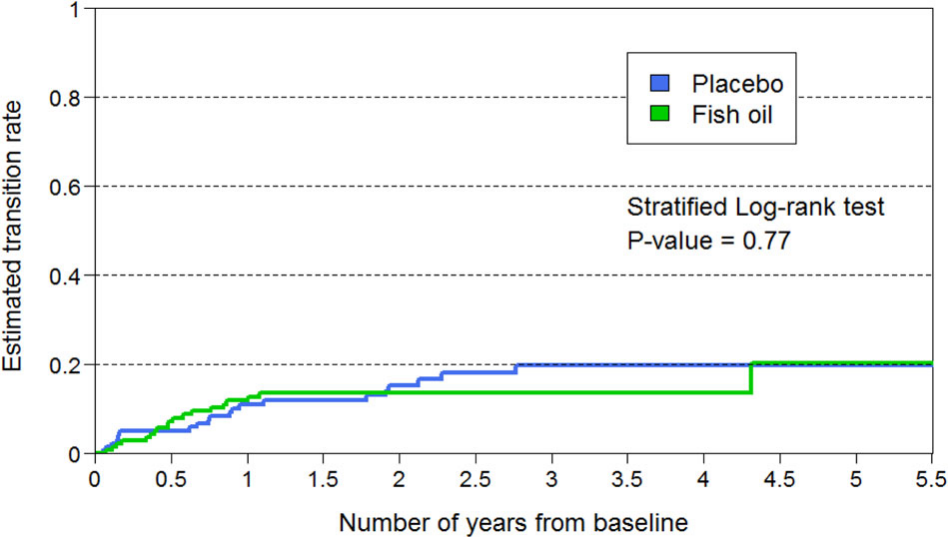
Survival curves showing transition to psychosis in the two treatment groups

The survival curves comparing transition rates of the two treatment groups are shown in Fig. 2. A stratified log-rank test was conducted on these survival curves, adjusting for the effects of the two stratifying factors (site and baseline MADRS score). Site had three levels (Melbourne: *n* = 106, Vienna: *n* = 72, and other sites: *n* = 126). The baseline MADRS score was divided into two levels in randomisation:<21 and ≥21. The stratified log-rank test indicated that there was no significant difference between the two treatment groups in transition rates (*p* = 0.77). Cox regression was also used to compare the transition rates with site and MADRS score as stratifying variables. The hazard ratio of omega-3 PUFA vs. placebo was 0.91 (95% CI = 0.41–1.70), *p* = 0.77.

#### Secondary outcome measures

General linear model analysis was used to compare the two treatments on symptom and functioning measures at medium-term follow-up with the corresponding baseline score, site and baseline MADRS score as covariates. As the follow-up time had a substantial range, its effect was accounted for as a factor of three levels (≤3 years, 3–4 years, and >4 years from baseline). In order to examine whether the difference between the two treatments varied between the different levels of follow-up time, the interaction between follow-up time and treatment was also analysed.

All symptom and functioning measures showed improvement between baseline and medium-term follow-up (Table 1). The *p*-values for the interaction term (*p*-value 1) of all the measures were non-significant, indicating that the difference between the two treatments did not vary between different follow-up times. The *p*-values for treatment (*p*-value 2) were also non-significant for all measures, indicating that the two treatments did not differ in their effects. Approximately 50% of cases for whom data were available (*n* = 166) had remitted from UHR status, with no difference in remission rates between the treatment groups (49.4% in the omega-3 PUFA condition, 53.2% in the placebo condition; *p* = 0.44).

**Table 1:** General linear model analysis comparing placebo and omega-3 PUFA groups in terms of change between baseline and follow-up after adjusting for baseline score, site, and baseline MADRS score

	*n*	Baseline (mean)	Medium-term follow-up (mean)	Medium-term—BL change (mean)	Medium-term—BL change (SD)	*p*-value 1	*p*-value 2
CAARMS unusual thought content							
Placebo	77	5.6	1.9	−3.8	4.1	0.101	0.638
Omega-3 PUFA	84	4.7	1.4	−3.2	4.5		
CAARMS non-bizarre ideas							
Placebo	77	6.7	3.0	−3.7	3.9	0.356	0.788
Omega-3 PUFA	83	7.2	3.0	−4.1	4.5		
CAARMS perceptual abnormalities							
Placebo	77	6.5	3.2	−3.3	3.7	0.972	0.370
Omega-3 PUFA	84	6.3	2.8	−3.5	3.4		
CAARMS disorganised speech							
Placebo	77	3.5	0.9	−2.5	3.2	0.705	0.646
Omega-3 PUFA	82	3.7	1.1	−2.6	3.9		
BPRS total							
Placebo	70	41.4	32.1	−9.3	10.0	0.348	0.861
Omega-3 PUFA	71	41.6	32.3	−9.4	10.1		
BPRS psychotic subscale							
Placebo	76	8.5	5.3	−3.2	3.4	0.253	0.871
Omega-3 PUFA	76	8.2	5.3	−2.9	2.9		
BPRS anxiety							
Placebo	76	3.5	2.1	−1.4	1.7	0.862	0.816
Omega-3 PUFA	76	3.2	2.1	−1.1	1.6		
SANS							
Placebo	69	16.4	9.8	−6.6	12.3	0.931	0.495
Omega-3 PUFA	71	18.5	11.5	−7.1	12.3		
YMRS							
Placebo	70	2.7	1.9	−0.8	2.8	0.572	0.286
Omega-3 PUFA	71	3.2	1.5	−1.7	3.7		
MADRS							
Placebo	75	19.5	9.8	−9.7	10.5	0.589	0.663
Omega-3 PUFA	76	20.6	10.9	−9.7	10.4		
SOFAS							
Placebo	89	54.1	69.4	15.3	16.5	0.366	0.518
Omega-3 PUFA	91	52.7	67.5	14.8	18.3		
Global functioning—social scale							
Placebo	76	6.6	7.4	0.8	1.6	0.104	0.483
Omega-3 PUFA	76	6.6	7.2	0.6	1.4		
Global functioning—role scale							
Placebo	76	6.0	7.3	1.3	1.7	0.468	0.385
Omega-3 PUFA	76	6.0	7.1	1.0	1.6		

*n* number with baseline and medium-term follow-up data, *BL* baseline, *SD* standard deviation of change score, *p-value 1* *p*-value for the interaction between follow-up time and treatment, *p-value 2* *p*-value for treatment, *CAARMS* Comprehensive Assessment of At-Risk Mental State, *BPRS* Brief Psychiatric Rating Scale, *SANS* Scale for the Assessment of Negative Symptoms, *MADRS* Montgomery-Asberg Depression Rating Scale, *YMRS* Young Mania Rating Scale, *SOFAS* Social and Occupational Functioning Scale, *PUFA* polyunsaturated fatty acids

*Note*: CAARMS scales were calculated as the sum of the intensity and frequency scales

Most improvement on the symptom and functioning measures appeared to have been achieved by end of the intervention period (12 months), with only minimal further improvement between 12-month and medium-term follow-up. For example, the mean BPRS score across both treatment groups reduced by 7.8 (SD = 8.9) points by 12 months and by a further 1.5 by points by medium-term follow-up; mean SANS score reduced by 6.9 (SD = 11.7) points by 12 months and the same at medium-term follow-up; mean MADRS score reduced by 9.3 points (SD = 9.5) by 12 months and by a further 0.4 points at medium-term follow-up; mean SOFAS score increased by 14.5 points (SD = 18) by 12 months and by a further 0.6 points by medium-term follow-up.

### Baseline predictors of transition to psychosis

The list of potential predictors included demographic variables, symptom and functioning measures, and recruiting site. As in the other analyses, recruiting site was treated as a factor of three levels (Melbourne, Vienna, Other sites) with Melbourne used as the reference level. The association between each variable and transition risk was tested individually using Cox regression (Table 2).

**Table 2:** Cox regression of the association between transition to psychosis and each baseline variable individually

	*n*	*β*	se (*β*)	*p*-value
Age	304	0.044	0.032	0.168
Gender	304	0.017	0.317	0.957
Ethnicity (Caucasian vs. non-Caucasian)	297	0.367	0.381	0.336
Years of education	297	−0.033	0.031	0.296
DUS	284	0.00016	0.00012	0.175
Log (DUS)	284	−0.034	0.104	0.747
CAARMS unusual thought content	304	0.024	0.044	0.587
CAARMS non-bizarre ideas	304	−0.017	0.049	0.725
CAARMS perceptual abnormalities	303	−0.015	0.059	0.794
CAARMS disorganised speech	299	0.109	0.051	0.033
BPRS total	296	0.061	0.013	0.000005
BPRS psychotic subscale	294	0.134	0.062	0.030
BPRS anxiety	296	0.354	0.107	0.00096
SANS total	295	0.035	0.010	0.001
SANS affective flattening or blunting	295	0.069	0.023	0.003
SANS alogia	295	0.107	0.048	0.024
SANS avolition-apathy	295	0.142	0.053	0.008
SANS anhedonia-asociality	295	0.076	0.036	0.038
SANS attention	294	0.166	0.086	0.054
YMRS total	294	0.032	0.053	0.547
MADRS total	304	0.024	0.018	0.187
SOFAS	298	−0.023	0.013	0.072
Global functioning—social scale	296	−0.353	0.124	0.004
Global functioning—role scale	296	−0.064	0.101	0.526
Site: Vienna vs. Melbourne	304	−0.703	0.521	0.177
Site: Other sites vs. Melbourne	304	0.433	0.349	0.216
Migrant status (yes vs. no)	297	−0.662	0.479	0.167

*n* number with baseline and medium-term follow-up data, *DUS* duration of untreated symptoms prior to study entry, *CAARMS* Comprehensive Assessment of At-Risk Mental State, *BPRS* Brief Psychiatric Rating Scale, *SANS* Scale for the Assessment of Negative Symptoms, *MADRS* Montgomery-Asberg Depression Rating Scale, *YMRS* Young Mania Rating Scale, *SOFAS* Social and Occupational Functioning Scale. Sub-scales are also reported when total scale scores were significant predictors

A stepwise cox regression was then performed to analyse the predictive value of the variables after adjusting for each other. This analysis resulted in total BPRS score (*p* = 0.0000002), ethnicity (*p* = 0.002), and migrant status (*p* = 0.033) being selected as independent predictors of transition, with CAARMS Disorganised Speech, BPRS Psychotic sub-scale, BPRS Anxiety, SANS total, and SANS sub-scales, Global Functioning (Social) score no longer remaining significant predictive variables. The finding of ethnicity and migrant status as predictive seemed to be driven by higher transition risk among non-Caucasian, non-migrants. This group showed a higher hazard ratio for transition to psychosis than Caucasian non-migrants (HR = 5.98, 95% CI for HR = 2.08–16.61), Caucasian migrants (10.62, 95% CI for HR = 2.04–55.25), and non-Caucasian migrants (HR = 5.08, 95% CI for HR = 1.22–21.19). This finding seems to be accounted for by the fact that the sites most highly represented by non-Caucasian non-migrants (Hong Kong and Singapore sites) had a somewhat higher transition rate (16%) than the other sites over the medium term. However, the strength and precision of this finding is limited by the small sample sizes of these sites, as indicated by the wide 95% CIs.

### Baseline predictors of functioning at medium-term follow-up

A general linear model was used to test the association between each baseline variable and functioning (SOFAS score) at follow-up (see Table 3). The same variables were included as above, as well as time to follow-up (treated as a factor of three levels: ≤3 years, 3–4 years, and >4 years from baseline).

**Table 3:** General linear model analysis results for the association between SOFAS score at medium-term follow-up and each baseline variable individually

	*n*	*β*	se (*β*)	*p*-value
Age	180	0.072	0.265	0.785
Gender	180	−0.202	2.377	0.932
Race (Caucasian vs. non-Caucasian)	180	1.306	3.609	0.718
Years of education	180	−0.059	0.237	0.806
DUS	175	−0.002	0.001	0.028
Log(DUS)	175	−0.963	0.767	0.211
CAARMS unusual thought content	180	−0.141	0.330	0.670
CAARMS non-bizarre ideas	180	−0.091	0.384	0.814
CAARMS perceptual abnormalities	179	−0.085	0.499	0.865
CAARMS disorganised speech	176	−0.140	0.375	0.709
BPRS total	180	−0.509	0.115	0.00002
BPRS psychotic subscale	179	−1.101	0.434	0.012
BPRS anxiety subscale	180	−2.954	0.729	0.00008
SANS total	179	−0.308	0.083	0.000
SANS affective flattening or blunting	179	−0.312	0.206	0.132
SANS alogia	179	−0.582	0.395	0.143
SANS avolition-apathy	179	−1.726	0.364	0.000004
SANS anhedonia-asociality	179	−0.911	0.265	0.0007
SANS attention	179	−3.122	0.636	0.000002
YMRS	180	−1.014	0.421	0.017
MADRS	180	−0.517	0.128	0.00008
Global functioning—social scale	180	3.606	0.897	0.00009
Global functioning—role scale	180	3.025	0.766	0.0001
Site: Vienna vs. Melbourne	180	11.319	2.807	0.00008
Site: Other sites vs. Melbourne	180	8.121	2.672	0.003
Follow-up time: 3–4 vs. ≤3 years	180	−2.228	2.710	0.412
Follow-up time: >4 vs. ≤3 years	180	−5.846	3.026	0.055
Migrant status (yes vs. no)	180	−0.862	2.870	0.764

*n* number with baseline and medium-term follow-up data, *DUS* duration of untreated symptoms prior to study entry, *CAARMS* Comprehensive Assessment of At-Risk Mental State, *BPRS* Brief Psychiatric Rating Scale, *SANS* Scale for the Assessment of Negative Symptoms, *MADRS* Montgomery-Asberg Depression Rating Scale, *YMRS* Young Mania Rating Scale, *SOFAS* Social and Occupational Functioning Scale. Sub-scales are also reported when total scale scores were significant predictors

A stepwise procedure was again used to choose predictive baseline variables after adjusting for each other. The variables identified were BPRS Anxiety subscale, the Global Functioning (Role) score, SANS Attention subscale and site (eTable 4), with the BPRS psychotic subscale, YMRS, MADRS, and Global Functioning (Social) score no longer remaining as significant predictors. The Melbourne site showed lower follow-up SOFAS scores than the Vienna site (11 points) and other sites (8.6 points). The model using these three variables explained 25.3% of the variation in SOFAS scores.

### Baseline and end of treatment (12-month) clinical measures in relation to medium-term follow-up

The correlations of clinical measures between medium-term follow-up and baseline and end of treatment (12 month) are presented in eTable 5. On the whole, both sets of correlations were moderate in magnitude, with the 12-month medium-term follow-up correlations stronger than the baseline medium-term follow-up correlations. This indicates that symptomatology and functioning levels at the end of treatment were a reasonably good indicator of the same several years later.

## Discussion

This paper reports a medium-term follow-up of an intervention trial of omega-3 PUFA plus CBCM vs. placebo plus CBCM in a UHR sample. The findings indicated a non-trivial but modest increase in proportion of known transitions between 12-month and medium-term follow-up (11–13%), with no significant difference in transition rate between the two treatment groups. On average, participants improved in their clinical symptomatology/functioning between baseline and medium-term follow-up, regardless of treatment condition, and approximately half remitted from UHR status. In fact, the group-level improvement was substantial, with clinical ratings indicating improvement from moderate/serious symptomatology and functional impairment at entry to mild symptoms and functional impairment at follow-up. On the whole, this improvement appeared to have been achieved by the end of the intervention period (12 months), with only minor further improvement between 12-month and medium-term follow-up.

The strongest independent baseline predictor of transition to psychosis was general psychopathology levels (total BPRS score). Participants of a non-Caucasian non-migrant background had a somewhat higher transition risk, which seemed to be a site effect. Baseline anxiety, negative symptoms (particularly attention disturbances), and poor role functioning independently predicted poor functional outcome. A further site effect was also apparent, with participants from the Melbourne site showing substantially poorer functioning at medium-term follow-up.

The modest increase in transition rate after cessation of specific targeted intervention is consistent with some previous medium-term follow-up studies.^18,27^ However, it is inconsistent with other studies^15,24,26^ which reported a substantial escalation in transition rate after end of treatment and concluded that the benefits of specific intervention were only apparent during the treatment phase. There are a number of possible reasons for the only modest increase observed in the current study. All participants received CBCM during the intervention phase. The CBT aspect of this intervention was specifically directed toward cognitive restructuring, building resilience against stress, and stress management.^23^ These aspects of the psychotherapy may have been protective over the longer term, rather than just dealing with immediate mental health and contextual difficulties during treatment. This is consistent with the higher medium-term transition rate in the Hong Kong and Singapore sites compared to the other sites, because these sites provided a lower number of CBCM sessions (mean of 7.2 sessions compared to 10.6 sessions among the other sites). The possibility of the effect of CBCM, with omega-3 PUFAs not conferring additional benefits, is indicated by the lack of difference in transition rate between the treatment groups at 12-month and medium-term follow-up, as well as by the substantial clinical improvements achieved in both groups by the end of the intervention phase.

Approximately half (55%) of the sample for whom data were available received ongoing mental health treatment after the end of the study intervention period, which may have had the effect of maintaining the gains achieved during the intervention phase as well as preventing deterioration. A substantial proportion of the sample (62%) received antidepressants during the intervention phase, which may had both an immediate effect on lowering the transition rate (improving mood and thereby indirectly reducing faulty appraisal of anomalous experiences linked to future psychosis^28^) and conferred benefit over the longer term (modulating response to environmental stress/adversity^29,30^ and possibly also via a neuroprotective route^31–33^). The use of antidepressants in approximately 20% of participants over the follow-up period may also have contributed to preventing the transition rate from escalating more substantially over this period, as well as possibly also contributing to the ongoing improvement in psychopathology and functioning. Additionally, approximately 10% of the sample used antipsychotic medication over the follow-up period (although this was mainly in participants who transitioned to psychosis). Finally, the use of omega-3 supplementation or change in dietary habits was not controlled after the study intervention period, which may have contributed to lack of difference between the treatments groups at medium-term outcome and the modest overall transition rate, if indeed omega-3 PUFAs do have a protective effect in high-risk patients. Although most clinical improvement seemed to have been achieved by the end of the intervention period (12 months), the high proportion of the sample who received (psychosocial and pharmacological) mental health support after the intervention period indicates ongoing clinical need in this population after 1 year of care.

The possibility of this sample not being sufficiently enriched for psychosis risk must also be considered. In other words, it is possible that the modest transition rate was not due to the reasons outlined above, but may have been observed regardless of the type of treatment provided, or indeed whether treatment was provided or not. Earlier intervention,^34^ pre-screening characteristics such as referral pathways and demographic characteristics,^35–37^ and changing symptom profiles in cohorts^38^ have all been reported as playing contributing roles to the lower transition rate in recently recruited UHR cohorts.

The prediction analysis conducted in this report reaffirms^39^ the importance of time period of observation in determining predictor variables/models. Specifically, different baseline variables predicted short (12-month) vs. medium-term (>2 year) outcome in this sample. Baseline depression was a strong predictor of transition by 12 months,^22^ with a MADRS cut-off score of 14 distinguishing between a 16.5 vs. 0% 12-month transition rate, whereas depression did not appear as a significant predictor over the medium term, with general psychopathology scores playing a stronger predictive role. This suggests, from a clinical point of view, that inferring level of risk based on clinical characteristics of new patients should not be based solely on research into predictors of short-term outcome, but also on variables that predict the longer-term outcome of patients. For example, if results from the current cohort were to be used to form a clinical risk calculator,^40^ entry depression scores might have identified those at short-term risk but missed those at medium-term risk. The results also show, as one would expect, that clinical state over the medium term appears to be better predicted by clinical state at the end of 12 months of treatment than by baseline symptom and functioning scores.

Baseline anxiety, poor role functioning, and negative symptoms (particularly attention disturbances) predicted poor functional outcome. The predictive role of negative symptoms is consistent with previous findings in UHR cohorts.^39,41–43^ While anxiety is known to be associated with compromised functioning in adult populations,^44,45^ its role may have been somewhat neglected in psychosis risk research and youth mental health more broadly. The predictive role of anxiety, general psychopathology, depression (over the short term) and negative symptoms for poor outcome (psychosis onset and poor functioning) reinforces the value of a broad, formulation-based target of intervention for the UHR group.^46^ Although attenuated positive psychotic symptoms are a common feature of the population (apart from the small minority only meeting transient psychosis or genetic risk criteria), a range of general psychopathological characteristics play a role in predicting poor outcome and should therefore be a focus of intervention.

Functioning levels at medium-term outcome were found to vary by recruitment site, with the Melbourne site showing poorer functioning than other sites. This may have been due to socio-economic factors, given that a large portion of the Melbourne site’s catchment area is of deprived socio-economic status compared to the other recruiting sites. This differential outcome by site does not seem to have been due to differences in treatment received over the follow-up period, with 55–60% of participants at each recruitment site receiving treatment over this period.

Given the negative findings of this trial, future work needs to further investigate whether omega-3 PUFA may play a role in treatment of this patient population. Other trials are currently underway to this end (https://clinicaltrials.gov/ct2/show/NCT01429454; https://clinicaltrials.gov/ct2/show/record/NCT02597439). The efficacy of omega-3 PUFAs in sub-groups of patients should also be investigated—for example, in those with aberrant membrane fatty acid levels or inflammatory markers. Predictive modelling in this patient population should also incorporate time-dependent or dynamic characteristics,^47,48^ rather than relying solely on baseline clinical variables, and examine predictors of persistent or incident non-psychotic outcomes.^49,50^ Group-level prediction analysis, as we have conducted in the current report, may mask important sub-group (e.g., “poor” vs. “good” outcome) differences, which will be the focus of subsequent reports. Finally, although a high follow-up rate (89%) was achieved on the main outcome of interest (transition to psychosis), availability of follow-up data on other outcomes (59% for functioning and 41–46% for symptom measures) were more modest. This may have introduced an attrition bias toward more favourable outcome data, given that previous research^51^ indicates difficulty recontacting members of adolescent and young adult psychiatric cohorts is associated with increased presence of disorder at follow-up.

## Conclusion

This medium-term follow-up indicated substantial improvement in symptoms and functioning in a UHR cohort over a mean 3.4-year follow-up period, with no difference between the omega-3 and placebo-treated groups. Most of this improvement had been achieved by the end of the intervention period (12 months), although high rates of post-intervention mental health service use indicate ongoing clinical need after this time. This post-intervention phase intervention or the longer-term effect of CBCM, or a combination of the two, may have contributed to maintaining the gains achieved during the intervention phase and prevented significant deterioration after this time.

## Method

### Study design

This was a randomised, double-blind, placebo-controlled treatment trial of omega-3 PUFA plus CBCM or placebo plus CBCM. Treatment was provided for 6 months, with participants receiving further CBCM^52^ on the basis of need between months 6 and 12. See Markulev et al.^23^ for full details of study methodology, inclusion/exclusion criteria and interventions, and McGorry et al.^22^ for the 6 and 12-month results.

The study was performed in accordance with the Declaration of Helsinki.^53^ The National Health and Medical Research Council of Australia (NHMRC) National Statement on Human Research was adhered to and appropriate ethical approval was obtained by each site (Melbourne, Australia: Melbourne Health Research Ethics Committee; Sydney, Australia: Sydney South West Area Health Service Ethics Review Committee; Basel, Switzerland: Ethics Commission for Basel; Zurich, Switzerland: Cantonal Ethics Commission Zurich; Jena, Germany: University Clinic Jena Ethics Commission; Copenhagen, Denmark: Capital Region Research Ethics Committee; Hong Kong: Institutional Review Board of the University of Hong Kong/Hospital Authority Hong Kong West Cluster; Vienna, Austria: Medical University of Vienna Ethics Commission; Singapore: National Healthcare Group Domain Specific Review Board; and Amsterdam, the Netherlands: Academic Medical Centre Medical Ethics Committee). Written informed consent was obtained for those younger than 17 years, parental or guardian consent was sought. The trial was registered at the Australia and New Zealand Clinical Trials Registry (ID 12608000475347).

### Medium-term follow-up

Medium-term follow-up of the sample was conducted in 2015–2016. The procedure consisted of the following steps to locate and recontact participants: (1) accessing the National Death Index to determine whether any participant had died since last contact, (2) research files, (3) public mental health service record systems, (4) National Electoral Roll, (5) telephone directory, (6) previous contacts, and (7) internet-based searching. Re-contacted participants were invited to a comprehensive face-to-face interview. If individuals did not consent to face-to-face assessment, they were asked for a brief telephone assessment, enabling collection of a minimum dataset. The brief assessment consisted of a sub-set of measures, including determination of transition status and functioning levels. When participants could not be contacted, hospital records were consulted to collect details of public health service contact, date of contact and diagnosis.

### Outcome measures

The main outcome of interest was transition to psychosis, with transition defined on the basis of operationalized criteria and assessed with the Comprehensive Assessment of the At-Risk Mental State (CAARMS).^42^ Diagnoses were determined with the Structured Clinical Interview for DSM-IV-TR Axis I Disorders. If CAARMS data were not available, the public mental health records were accessed to determine if the participant had developed a psychotic disorder. Secondary outcome measures included the Brief Psychiatric Rating Scale (BPRS),^54^ the Scale for the Assessment of Negative Symptoms (SANS),^55^ the MADRS,^56^ the Young Mania Rating Scale (YMRS),^57^ the SOFAS,^58^ and the Global Functioning: Social^59^ and Role^60^ scales.

### Statistical analysis

The primary analysis used the intention-to-treat approach, comparing the difference in transition rates between the treatment groups using survival analysis with the stratified log-rank test and Cox regression with recruitment site and baseline Montgomery-Asberg Depression Rating Scale (MADRS)^56^ score (<21 and ≥21) as stratifying factors. General linear modelling was used to compare the secondary outcomes between the two treatment groups. Baseline clinical predictors of outcome were examined using stepwise cox regression. The relationship between clinical measures at the end of the treatment phase and medium-term follow-up was examined using Pearson correlation.

### Data availability

The data that support the findings of this study are available from the corresponding author upon reasonable request.

## Electronic supplementary material

Supplementary material
