# Supplementary material for: NEURAPRO: a multi-centre RCT of omega-3 polyunsaturated fatty acids versus placebo in young people at ultra-high risk of psychotic disorders—medium-term follow-up and clinical course
Source: NPJ Schizophr. 2018 Jun 25;4:11. doi: 10.1038/s41537-018-0052-x (PMC6018097; doi:10.1038/s41537-018-0052-x)
Supplement: Supplementary file 1 — Supplementary material [file 41537_2018_52_MOESM1_ESM.pdf]

eTable 1

Numbers and percentages of participants assessed using each type of assessment in the medium term follow up

	Placebo		Omega-3 PUFA		All	
	Number	%	Number	%	Number	%
No assessment	14	9.3	20	13.1	34	11.2
Full assessment	76	50.3	76	49.7	152	50.0
Brief assessment	14	9.3	15	9.8	29	9.5
Hospital record	47	31.1	42	27.5	89	29.3
Total	151	100	153	100	304	100

Notes.
PUFA = polyunsaturated fatty acids

eTable 2
Percentage of cases recorded to have used medications between end of the intervention period and the medium term follow-up

	Placebo (n=137)	Omega-3 PUFA (n=133)	p-value*
Antidepressant	21.2	18.0	0.543
Anti-inflammatory	1.5	1.5	>0.999
Antipsychotic	11.7[+]	9.8[+]	0.696
Mood stabiliser	1.5	1.5	>0.999
Sedative	2.9	3.8	0.747
Stimulant	0.7	0	>0.999
Tranquilizer	4.4	6.0	0.593
Other	13.9	9.8	0.349

Notes.
PUFA = polyunsaturated fatty acids
* Fisher exact test p-value comparing the two treatment groups.
[+] 11 of 16 cases (69%) in the placebo group prescribed antipsychotic medication and 7 of 13 cases (54%) in the omega-3 PUFA group prescribed antipsychotic medication were transitioned cases.

eTable 3

Number of cases who transitioned to psychosis by treatment group and time of follow up

Time of follow up	Placebo	Omega-3 PUFA	All
0-1 year	14	16	30 (75%*)
1-2 years	4	2	6 (15%)
2 years+	3	1	4 (10%)
Total	21	19	40 (100%)

Notes.
PUFA = polyunsaturated fatty acids
* Percentage values refer to percentage of total number of transitioned cases

eTable 4

Variables chosen by stepwise procedure as predictors of functioning (SOFAS score) at medium term follow up.

	p-value	β	se(β)
BPRS Anxiety sub-scale	0.018	-1.68	0.70
Global Functioning Score (Role)	0.0005	2.68	0.75
SANS Attention sub-scale	0.012	-1.69	0.67
Site: Vienna vs. Melbourne	0.005	8.38	2.96
Site: Other sites vs. Melbourne	0.014	6.38	2.57

Notes.
BPRS=Brief Psychiatric Rating Scale. SANS=Scale for the Assessment of Negative Symptoms.

eTable 5.
Correlations of clinical measures between baseline and medium term follow up and between end of treatment (month 12) and medium term follow-up.

	**Baseline and medium term follow up**			**Month 12 and medium term follow up**		
	Pearson correlation	p-value	n	Pearson correlation	p-value	n
BPRS Total	0.47	<0.001	141	0.57	<0.001	127
BPRS Psychotic subscale	0.27	0.001	152	0.61	<0.001	138
BPRS Anxiety	0.27	0.001	152	0.30	<0.001	139
SANS Total	0.57	<0.001	140	0.63	<0.001	128
SANS Affective flattening or blunting	0.46	<0.001	139	0.49	<0.001	127
SANS Alogia	0.44	<0.001	151	0.38	<0.001	139
SANS Avolition-Apathy	0.45	<0.001	151	0.60	<0.001	139
SANS Anhedonia-Asociality	0.54	<0.001	151	0.67	<0.001	139
SANS Attention	0.44	<0.001	151	0.56	<0.001	139
YMRS	0.28	0.001	141	0.21	0.020	124
MADRS	0.40	<0.001	151	0.60	<0.001	138
SOFAS	0.22	0.004	180	0.58	<0.001	157
Global Functioning – Social Scale	0.35	<0.001	152	0.36	<0.001	139
Global Functioning – Role Scale	0.29	<0.001	152	0.36	<0.001	139

Notes.
BPRS=Brief Psychiatric Rating Scale. SANS=Scale for the Assessment of Negative Symptoms. MADRS= Montgomery-Asberg Depression Rating Scale. YMRS=Young Mania Rating Scale. SOFAS=Social and Occupational Functioning Scale.
